# Standardization of SARS-CoV-2 Nucleic Acid Amplification Techniques by Calibration and Quantification to the First WHO International Standard for SARS-CoV-2 RNA

**DOI:** 10.1155/2023/7803864

**Published:** 2023-02-17

**Authors:** Jolanda J. C. Voermans, Daphne G. J. C. Mulders, Rob J. J. Beerkens, Marlize van Duijn, Liesbeth van der Zwaan, Janette Rahamat-Langendoen, Annemiek van der Eijk, Marion P. G. Koopmans, Richard Molenkamp

**Affiliations:** ^1^Department of Viroscience, Erasmus Medical Center, Rotterdam, Netherlands; ^2^Department of Microbiology, Erasmus Medical Center, Rotterdam, Netherlands

## Abstract

Clinical decision-making regarding isolation of SARS-CoV-2 patients is usually based on semiquantitative cycle-threshold (Ct) values without standardization. However, not all molecular assays produce Ct values, and there is ongoing discussion about whether Ct values can be safely used for decision-making. In this study, we standardized two molecular assays which use different nucleic acid amplification techniques (NAAT): the Hologic Aptima SARS-CoV-2/Flu (TMA) and Roche Cobas 6800 SARS-CoV-2 assays. We calibrated these assays against the first WHO international standard for SARS-CoV-2 RNA by using linear regression of log10 dilution series. These calibration curves were used to calculate viral loads for clinical samples. Clinical performance was assessed retrospectively using samples collected between January 2020 and November 2021, including known positives of the wild-type SARS-CoV-2 virus, the VOCs (alpha, beta, gamma, delta, and omicron) and quality control panels. Linear regression and Bland–Altman analysis showed good correlations for SARS-CoV-2 between Panther TMA and Cobas 6800 when standardized viral loads were used. These standardized quantitative results can benefit clinical decision-making and standardization of infection control guidelines.

## 1. Introduction

Since the beginning of the SARS-CoV-2 pandemic in 2019, over 575 million confirmed cases and over 6.3 million deaths have been reported worldwide (WHO Coronavirus (COVID-19) Dashboard, 2 August 2022). The burden of disease resulted in reorganization of hospitals in order to care for COVID-19 patients, and in many hospitals, the treatment of other diseases was downscaled and noncritical care was limited to urgent cases only [1]. From the beginning of the pandemic, the gold standard for detection of SARS-CoV-2 infection has been nucleic acid amplification techniques (NAAT) such as RT-PCR. A complicating factor has been the observation that individuals can remain PCR-positive for a considerable amount of time, even when the patient has recovered and symptoms have disappeared [2]. Studies early in the pandemic showed that prolonged RT-PCR positivity did not correlate with presence of the infectious virus [3, 4], which led to the practice of using semiquantitative RT-PCR results, such as cycle-threshold (Ct) values to guide infection prevention measures and release of patients from isolation [5]. Since Ct values can differ significantly between different tests and laboratories, there is ongoing discussion whether Ct values can be reliably used to set clinical cutoffs [6]. In addition, other NAATs such as transcription mediated amplification (TMA) and loop-mediated isothermal amplification (LAMP) do not provide semiquantitative values. The Hologic Aptima SARS-CoV-2/Flu assay combines the technologies of target capture and real-timetranscription-mediated amplification (TMA) and produces “time to positivity” (TTP) measurements that in principle could be used as semiquantitative results. We have standardized the Hologic Aptima SARS-CoV-2/Flu and Roche Cobas 6800 SARS-CoV-2 assays for detection of SARS-CoV-2 by calibrating these assays to the first international standard for SARS-CoV-2 RNA and have evaluated the quantitative results.

## 2. Materials and Methods

Analytical sensitivity of both assays was performed using log10 dilution series (1.00E6–1.00E0 IU/mL) of the first WHO international standard for SARS-CoV-2 RNA (NIBSC 20/146, 7.70 log10 IU/mL). All dilution series were prepared in Dulbecco's modified Eagle medium (DMEM) with 4.5 g/l glucose (Lonza, BESP070F), aliquoted, and stored at −80°C until used. Each dilution series was tested in 6 replicates on two different Panther systems in the SARS-CoV-2 Aptima SARS-CoV-2/Flu assay (Panther TMA) and in the Cobas 6800 SARS-CoV-2 assay [7, 8]. In addition, external quality assessment panels (EQAs) provided by the National Institute for Public Health and the Environment (RIVM; “EQA 20.4” and “sensitivity panel 20-03”) were tested.

Clinical performance was evaluated retrospectively with respiratory tract samples collected and stored at −80°C between January 2020 and November 2021. This included known positives of wildtype SARS-CoV-2 and the variants of concern (VOC) alpha, beta, gamma, delta, and Omicron. Due to logistic constraints, there was one additional freeze/thaw cycle before testing on the Panther TMA and GeneXpert systems, compared to Cobas 6800.

Pretreatment of sputa (SP) was performed as described before [9, 10]. Throat and nasal swabs were not pretreated. Samples (500 *μ*L) were added directly to the Panther Fusion Specimen Lysis tubes, containing 710 *μ*L specimen transport media (STM). According to the manufacturer, Panther TMA results are qualitative, but also time-to-positivity (TTP) results are reported as a semiquantitative measure. This is the time, given in minutes with one decimal after the decimal point, necessary for the fluorescent signal to reach a specified detection threshold. In this study, we have used these TTP results to correlate the measurements to the quantitative international WHO RNA standard. For the Cobas 6800 SARS-CoV-2 assay, samples were inactivated prior to the analysis by adding 500 *μ*L of the sample to 750 *μ*L MagnaPure 96 external lysis buffer (Roche) as per the routine protocol for SARS-CoV-2 detection in our laboratory. The Roche Cobas 6800 SARS-CoV-2 assay targets the ORF1a/b nonstructural region (target 1) and the pan-SarbecoE-gene region (target 2). Calibration to the quantitative international WHO RNA standard was performed with the Ct values of the ORF1a/b nonstructural region (target 1). Discrepant samples were retested in the Xpert Xpress SARS-CoV-2/Flu/RSV plus assay (GeneXpert, Cepheid) [11, 12].

Statistical analysis was performed using IBM SPSS v2.1, and 95% specified Clopper–Pearson confidence intervals for sample proportion were calculated using Epitools (https://epitools.ausvet.com.au).

## 3. Results

Analytical sensitivity of Panther TMA was 9.17E1 IU/mL (range 3.59E1–9.66E3), determined by probit analysis at the 95% hit rate, while analytical sensitivity of the Cobas 6800 ORF1a/b assay was 1.82E2 IU/mL (range 1.07E2–3.56E3), resulting in a difference of 0.30 log10. In the SARS-CoV-2 RIVM EQA panel, the Cobas 6800 ORF1a/b assay scored 100%. One sample was not detected by the Panther TMA assay, compared to the expected results, whereas the Cobas 6800 pan-Sarbeco assay failed to detect two samples. One additional sample was detected in the Cobas 6800 pan-Sarbeco assay which, according to the panel composition information, contained SARS-CoV-1.

The retrospective clinical SARS-CoV-2 study consisted of 60 clinical respiratory tract samples from 60 patients, of which 52 combined nasal/throat swabs (NTS), 7 sputa (SP), and 1 nasal swab (NS). Of these clinical samples, 51.7% (*n* = 31, 3 wildtype, 7 alpha, 2 beta, 1 gamma, 13 delta, and 5 omicron BA1 variants) was SARS-CoV-2 positive for both Panther TMA and Cobas 6800. This resulted in a clinical sensitivity and specificity of 100% and 93.1%, respectively (Table 1). Since there was no difference in clinical sensitivity and specificity between Cobas 6800 ORF1a/b and pan-Sarbeco results, only the Cobas 6800 ORF1a/b results were used for further analysis. Panther TMA detected SARS-CoV-2 in two additional samples (TTP 20.1, VOC delta and TTP 19.9, VOC omicron BA1). Both samples tested positive for the geneXpert assay (Ct: 35.6 and 36.5, respectively).

**Figure 1:**
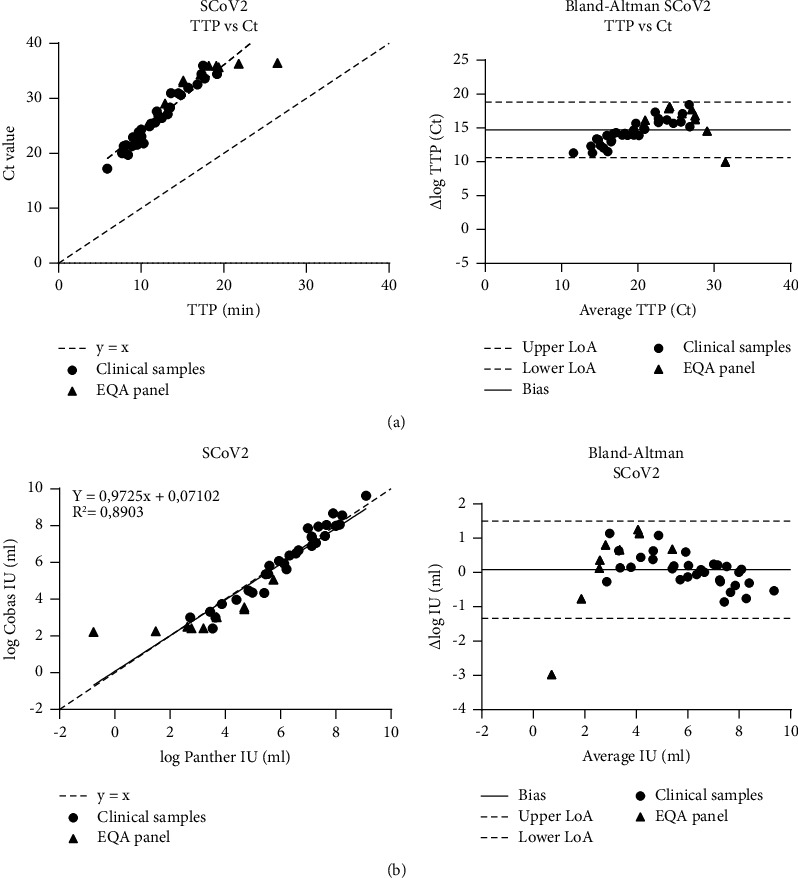
Correlation plot and Bland–Altman plot of paired Panther TMA TTP and Cobas 6800 ORF1a/b Ct values (a) and IU/ml values (b) for SARS-CoV-2 (SCoV2). The slope and bias show a significant deviation from zero when semiquantitative Ct and TTP values are used.

In Figure 1(a), Ct values from the Cobas 6800 ORF1a/b nonstructural region were plotted against the TTP values from the Panther TMA assay. Only samples positive for both the Cobas 6800 ORF1a/b and Panther TMA assays were included in the regression analysis. As expected, results showed a deviation from zero in both Deming regression and the Bland–Altman plot, confirming that these results cannot be compared without standardization. For standardization calibration, curves were constructed by linear regression for the Panther TMA (*y* = −2.0833, *x* + 24.897, and *R*^2^ = 0.9391) and Cobas 6800 ORF1a/b assays (*y* = −2.8507, *x* + 43.461, and *R*^2^ = 0.9782) using the results of the log10 dilution series from the internal standard. These calibration curves were used to calculate the viral loads of the samples from the clinical evaluation and the RIVM EQA panel in IU/ml. Deming regression analysis, when IU/ml values were used (Figure 1(b)), showed a good correlation (slope 0.9725, *R*^2^ = 0.8903) between the TMA and Cobas 6800 ORF1a/b assays. The Bland–Altman plot showed a bias of 0.08 with upper and lower limits of agreements (LoAs) of 1.50 and −1.34, respectively. This resulted in one outlier, a sample from the RIVM EQA sensitivity panel with a difference in the viral load between the Panther TMA and Cobas 6800 ORF1a/b assays of 2.98 log10 (TTP (26.5) and Ct value (36.4), respectively).

## 4. Discussion

There are limited studies that are routine SARS-CoV-2 molecular assays which are calibrated and standardized to international standards. Sahoo et al. described comparison of GeneXpert to a laboratory-developed test using the first WHO international RNA standard and showed harmonization and standardized quantification [13]. Using a similar approach, we were able to calibrate the semiquantitative measurements from the Panther Aptima SARS-CoV-2 real-time TMA and Cobas 6800 SARS-CoV-2 ORF1a/b assays to a relevant quantitative standard. Dilution series showed linear correlations with an analytical sensitivity of 9.17E1 IU/ml for Panther TMA and 1.82E2 for Cobas 6800 ORF1a/b assays, respectively. Overall, clinical sensitivity of Panther TMA was 100%, whereas the clinical specificity was 93.1% compared to that of the Cobas 6800 ORF1a/b and pan-Sarbeco assays. Since only two samples were positive for Panther TMA and negative for the Cobas 6800 ORF1a/b and pan-Sarbeco assays, this number should be taken with caution. Both samples were positive using the additional geneXpert assay; however, with high Ct values. This suggests a slightly higher clinical sensitivity for Panther TMA compared tothan for the Cobas 6800 ORF1a/b and pan-Sarbeco assays. However, additional analysis of samples with a low amount of the virus close to the detection limit of both assays is needed to draw more firm conclusions.

Deming regression and Bland–Altman analysis showed comparable results between the Panther TMA and Cobas 6800 ORF1a/b assays when calibrated IU/ml values were used. Noncalibrated values gave a deviation from zero, showing that the TTP and Ct values are not comparable and that calibration is necessary to gain reliable quantitative and comparable results.

Detection of SARS-CoV-2 can be performed on various sample types. The Panther TMA assay is intended for use with nasal and/or throat swabs only, the preferred sample type for SARS-CoV-2 detection at our hospital. However, for SARS-CoV-2 patients in the intensive care ward, sputa are also used. Pretreated samples of this sample type were included in the clinical study without problems.

Since the introduction of international standards in molecular diagnostics, laboratories have been able to produce reliable and comparable quantitative results between assays and laboratories, which opened the opportunity to international interlaboratory studies [14, 15]. Currently, quantitative molecular assays are fully integrated in the monitoring of infectious diseases. However, most quantitative assays have only been validated on homogeneous samples such as serum and plasma. Quantification of heterogeneous samples like respiratory tract samples is more challenging since also variations in sample composition, sample collection, and transport media can influence quantitative results. Although we report here that quantification to RNA standards is possible, we also stress caution with interpretation of these quantitative results due to the intrinsic variable nature of respiratory samples.

At the Erasmus Medical Centre, a tertiary care hospital, patients infected with SARS-CoV-2 are treated in one-person isolation rooms by healthcare workers using personal protective equipment. It is known that infection control measures can lead to decreased attention given to those who are treated in isolation and can have negative psychological effects on the patient. In our hospital, infection control measures for SARS-CoV-2 are lifted for patients admitted at the general ward after a minimum disease duration of 10 days, at least 2-3 days improvement of clinical symptoms and negative PCR or PCR with a high Ct value (Ct value > 32, [3]). Based on our studies, this Ct value is comparable with a viral load of <1.0E4 IU/ml. Following introduction of the calibrated quantitative real-time Panther TMA assays for SARS-CoV-2 in our laboratory, this viral load cutoff of 1.0E4 IU/ml is now used to guide clinical decision-making for infection control measures.

In conclusion, we were able to standardize the quantification of two molecular assays which are based on different detection systems by calibrating the results to the first international standard for SARS-CoV-2 RNA, which can lead to comparable results between assays and laboratories and can be helpful in clinical decision-making.

## Figures and Tables

**Table 1:** Clinical sensitivity and specificity of the Panther TMA assay compared to Cobas 6800 ORF1a/b and pan-Sarbeco assays.

	Frequency	Percentage	95% CI^*∗*^	95% CI^*∗*^
	(*n*)	(%)	Lower limit	Upper limit
Sensitivity	31/31	100.0	88.8	100.0
Specificity	27/29	93.1	77.2	99.2
NPV^#^	27/27	100.0	87.2	100.0
PPV^&^	31/33	93.9	87.2	100.0
Accuracy	58/60	96.7	88.5	99.6

^#^PPV = positive predictive value; ^&^NPV = negative predictive value; ^*∗*^95% CI = 95% confidence interval.

## Data Availability

All data used to support the findings of this study are available from the corresponding author on request.
